# Enhanced Diagnostic Accuracy of Pulmonary Embolism: Integrating Low-Dose CT with V/Q SPECT

**DOI:** 10.3390/tomography10080096

**Published:** 2024-08-16

**Authors:** Munassar Dakkam Lasloom, Mohamed Abuzaid

**Affiliations:** 1 Nuclear Medicine Department, King Khalid Hospital, Najran P.O. Box 1120, Saudi Arabia; mlasloom@moh.gov.sa; 2 Medical Diagnostic Imaging Department, College of Health Sciences, University of Sharjah, Sharjah 27272, United Arab Emirates

**Keywords:** pulmonary embolism, computed tomography, V/Q SPECT, diagnostic, SPECT

## Abstract

Objective: This study aimed to retrospectively assess the benefits of combining low-dose computed tomography (LDCT) with ventilation/perfusion single-photon emission computed tomography (V/Q SPECT) for the diagnosis of pulmonary embolism (PE). Methods: A retrospective analysis was performed on 92 patients with suspected PE who underwent V/Q SPECT with ldCT (V/Q SPECT CT) between January 2020 and December 2022 at King Khalid Hospital Najran. Data were collected using the hospital’s picture archiving and communication system. Scans were categorized on the basis of perfusion defects, matched or mismatched ventilation, and CT findings. The specificity of V/Q SPECT CT was compared with that of Q SPECT CT. Results: This study included 92 patients (54 females and 38 males; median age, 53 years). The results demonstrated that V/Q SPECT CT had higher specificity (93%) than V/Q SPECT alone (88%). If CT had been used as a ventilation substitute, 21% of patients would have been reported to be positive for PE (8% false-positive), yielding a specificity of 60% for Q SPECT CT. These findings align with the existing literature, although discrepancies in specificity values were noted due to the different study designs and sample sizes. Conclusion: This study highlights the enhanced specificity of V/Q SPECT CT compared to V/Q SPECT and Q SPECT CT alone. Including low-dose CT improves diagnostic accuracy by reducing false positives and providing detailed anatomical information. V/Q SPECT CT offers superior specificity in diagnosing PE compared with V/Q SPECT alone, supporting its use in clinical practice.

## 1. Introduction

Pulmonary embolism (PE) is a serious medical condition caused by a blood clot that blocks a blood vessel in a deep vein, typically in the legs, which can travel to the lungs and block an artery. This condition prevents the blood from reaching the lungs and other vital organs, making it potentially fatal. Diagnosis of PE is challenging because of its nonspecific symptoms and varying clinical presentations. Prevalence of PE is difficult to determine accurately due to inconsistent hospital records, but it affects an estimated 7 to 8 out of 10,000 people in the general population, with the incidence increasing with age. The risk factors for PE include long periods of inactivity, hospitalization, long-haul flights, obesity, smoking, hormone-based therapies, pregnancy, and cancer. PE is also more common in individuals with certain inherited conditions that increase the propensity for blood clotting [1,2].

Treatment for PE primarily involves anticoagulants, which prevent further clotting and allow the body to absorb the existing clots. However, anticoagulants carry the risk of severe bleeding, making accurate diagnosis crucial to avoid unnecessary treatment [3,4]. Traditional diagnostic methods for PE include D-dimer blood tests [5], ultrasonography, computed tomography pulmonary angiography (CTPA) [3,6,7], and lung ventilation/perfusion (V/Q) scans [3,8]. Although CTPA is widely regarded as the gold standard, it involves high radiation exposure and may not be suitable for all patients. Recent guidelines suggest that V/Q single-photon emission computed tomography (SPECT) could be a better first-line diagnostic tool because of its lower radiation dose and comparable accuracy [4,8].

V/Q SPECT, recommended by the European Association of Nuclear Medicine, offers high sensitivity and specificity but can produce false positives due to conditions other than PE causing V/Q mismatches [9]. Combining V/Q SPECT with low-dose computed tomography (ldCT) enhances diagnostic specificity by providing anatomical details that can help identify non-embolic causes of perfusion defects such as emphysema or pneumonia [3]. This combined approach is increasingly being recognized for its ability to improve diagnostic accuracy while minimizing radiation exposure. The transition from planar V/Q scintigraphy to SPECT has significantly advanced nuclear medicine, thereby improving diagnostic confidence and patient outcomes. Furthermore, ongoing research and systematic reviews continue to evaluate the effectiveness and safety of V/Q SPECT in clinical practice, aiming to solidify its role as a diagnostic algorithm for PE [3]. As evidence accumulates, it is crucial for clinical guidelines to adapt to ensure that patients receive the most accurate and safest diagnostic evaluations.

This study aimed to retrospectively assess the diagnostic benefits of combining low-dose computed tomography (ldCT) with ventilation/perfusion single-photon emission computed tomography (V/Q SPECT) for diagnosing pulmonary embolism (PE). The primary objective of this study was to determine whether integrating ldCT improves the diagnostic performance of V/Q SPECT by enhancing specificity and reducing false positives. The secondary objective was to evaluate whether ldCT could effectively substitute the ventilation component of V/Q SPECT. The novelty of this study lies in the investigation of a combined diagnostic approach that optimizes PE diagnosis, provides detailed anatomical information, and potentially improves patient care through more accurate and reliable detection.

## 2. Methods

### 2.1. Study Design

A retrospective analysis was performed on the data of 92 patients with suspected PE who had undergone V/Q SPECT with ldCT (V/Q SPECT CT) between January 2020 and December 2022 at the Nuclear Medicine Department of King Khalid Hospital Najran (KKHN). The study population was derived from a hospital’s picture archiving and communication system.

V/Q SPECT ldCT has been established as a routine diagnostic study for PE in nonpregnant patients at the hospital. Studies of suboptimal quality were excluded. All reports were obtained by two dual-trained nuclear medicine and radiology consultants.

The reports of all V/Q SPECT ldCTs were reviewed and categorized as follows.

1. Positive or negative perfusion defect.
2. The ldCT and ventilation scan findings were categorized as follows.

Category 1: CT and ventilation, both normal and matched.

Category 2: CT abnormal and normal ventilation (including incidental findings).

Category 3: CT normal and abnormal ventilation.

Category 4: CT and ventilation were both abnormal and matched.

### 2.2. Initial Ventilation/Perfusion Single-Photon Emission Computed Tomography: Interpretation, Diagnostic Management, and Follow-Up

All patients underwent V/Q SPECT, comprising perfusion and ventilation SPECT. Ventilation was performed using nebulized 99mTc-DTPA. Perfusion tests were performed using 99mTc-MAA at a standard dose of 200 MBq.

Approximately 1 GBq 99mTc-DTPA in 1–2 mL was placed in the 99mTc-DTPA aerosol delivery system (SmartVent™, Diagnostic Imaging Ltd., Northampton, UK). Then, 99mTc-DTPA was drawn from the reservoir by the action of a vibrating plate with 1000 precision-tapered holes to create aerosol drops, which the individual inhaled via a mouthpiece. The exhaled air was then confined within the filter attached to the aerosol unit, inhibiting the contamination of the equipment. Approximately 10% of the activity accumulated in the lungs of a typical patient, with the remaining airborne and exhaled. Deposition depends on aerosol particle size, shape, density, and electrical charge. Larger particles tend to settle in the central area, whereas smaller particles are deposited in the peripheral areas.

Images for analysis were collected using a dual-head camera equipped with low-energy, high-resolution collimators (Discovery NM/CT 670 Pro, GE Healthcare, Chicago, IL, USA). The images were acquired using energy windows centered on peaks of 140 keV ± 10%. The collected SPECT data were then corrected for attenuation. V/Q SPECT was interpreted by the nuclear medicine consultant in charge, using a diagnostic cut-off of one segmental or two subsegmental mismatched defects as per the EANM guidelines, considering the ldCT findings.

### 2.3. Low-Dose Computed Tomography Acquisition

All patients underwent V/Q SPECT scanning followed by an ldCT scan using the same SPECT-CT system (Discovery NM-CT 670; GE, California, CA, USA). The CT scan (110 kV, 16 mAs, pitch: 1.0) was conducted without contrast enhancement during free breathing directly after the initial V/Q SPECT scanning. Images were collected in the cephalocaudal direction using a 512 × 512 matrix. Axial, coronal, and sagittal slices were reconstructed in 1 mm thick sections. The protocol is summarized in Table 1.

There is a difference in the whole-body effective dose between males and females because of the radiosensitivity of various tissues. Females have more radiosensitive tissues, such as the breasts, resulting in a higher effective dose than males when scanned with V/Q SPECT CT. The GE Discovery CT scanner uses a dose-correcting algorithm, “OptiDose”, which attenuates CT X-rays more for large patients and less for small patients. The final exposure was adjusted between 0.68 and 0.86 mSv based on the individual.

### 2.4. Statistical Analysis

Statistical analysis was performed to evaluate the diagnostic accuracy of V/Q SPECT combined with low-dose CT for detecting pulmonary embolism (PE) across the study population. Descriptive statistics were used to summarize the demographic characteristics of the 92 patients, with the median age calculated and the distribution of sex analyzed. The diagnostic outcomes were categorized based on the presence or absence of perfusion and ventilation defects along with the corresponding CT findings. In addition, the sensitivity, specificity, and positive and negative predictive values of the combined V/Q SPECT-CT approach were calculated to quantify its diagnostic performance, with V/Q SPECT as the gold standard.

### 2.5. Ethical Approval

The Ministry approved this research study of Health Research Ethics Committees (Approval number: IRB Log Number 2020-13 E, 8 June 2023).

## 3. Results

### 3.1. Study Population

This study included 92 patients, comprising 54 females (60%) and 38 males (40%), aged between 18 and 94 years, with a median age of 53. All participants underwent high-quality V/Q SPECT CT.

### 3.2. Flowchart of V/Q SPECT and CT Findings in the Diagnosis of Pulmonary Embolism

**Figure 1:**
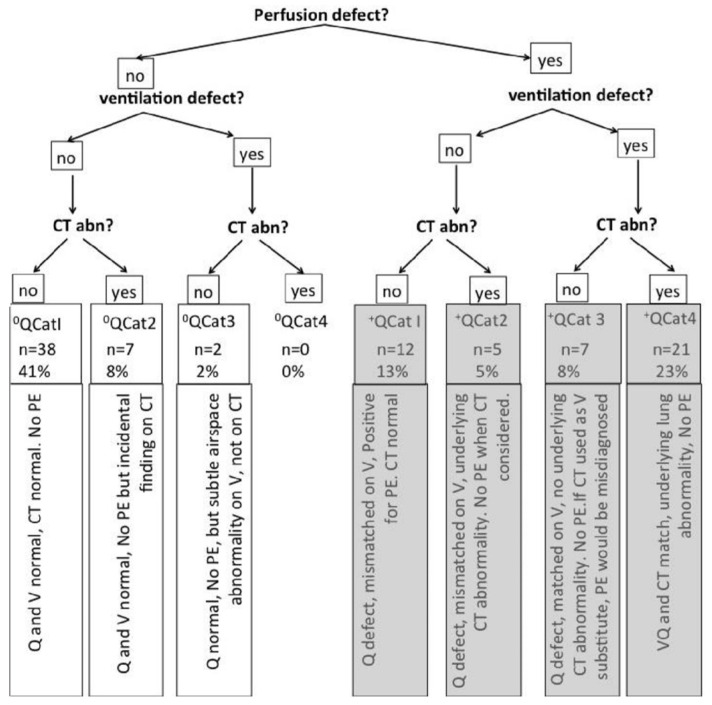
Flowchart of V/Q SPECT and CT findings for the diagnosis of pulmonary embolism.

Figure 1 shows a flowchart illustrating the diagnostic outcomes for patients suspected of pulmonary embolism (PE) using ventilation/perfusion (V/Q) single-photon emission computed tomography (SPECT) combined with low-dose computed tomography (CT). It begins by determining the presence of perfusion and ventilation defects and then assesses the CT scan findings.

For cases without a perfusion defect, if there was no ventilation defect and the CT was normal (0QCat1, *n* = 38, 41%), there was no PE. If the CT showed an incidental finding (0QCat2, *n* = 7, 8%), it also indicated no PE. When there was a ventilation defect but no CT abnormality (0QCat3, *n* = 2, 2%), there was no PE but a subtle airspace abnormality on V. There were no cases of either a ventilation defect or a CT abnormality without a perfusion defect (0QCat4, *n* = 0, 0%).

For cases with a perfusion defect, if there was no ventilation defect and the CT was normal (+QCat1, *n* = 12, 13%), it indicated PE. If the CT showed an abnormality (+QCat2, *n* = 5, 5%), there was no PE when CT was considered. When perfusion and ventilation defects are present without a CT abnormality (+QCat3, *n* = 7, 8%), it suggests no PE. However, PE can be misdiagnosed if CT is used as a substitute for V. Finally, if both perfusion and ventilation defects were present with a CT abnormality (+QCat4, *n* = 21, 23%), it indicated an underlying lung abnormality and no PE.

This flowchart shows how combining V/Q SPECT with CT enhances diagnostic accuracy by distinguishing PE from other conditions, thereby reducing the number of false positives.

### 3.3. Ventilation/Perfusion Single-Photon Emission Computed Tomography with Low-Dose Computed Tomography: Observations

A detailed examination of the CT scanning component is crucial for accurate interpretation. All scans were consistently of high quality and resolution, ensuring reliable data for analysis. The CT observations revealed significant insights into the conditions that might produce false positives in the V/Q SPECT results.

**Figure 2:**
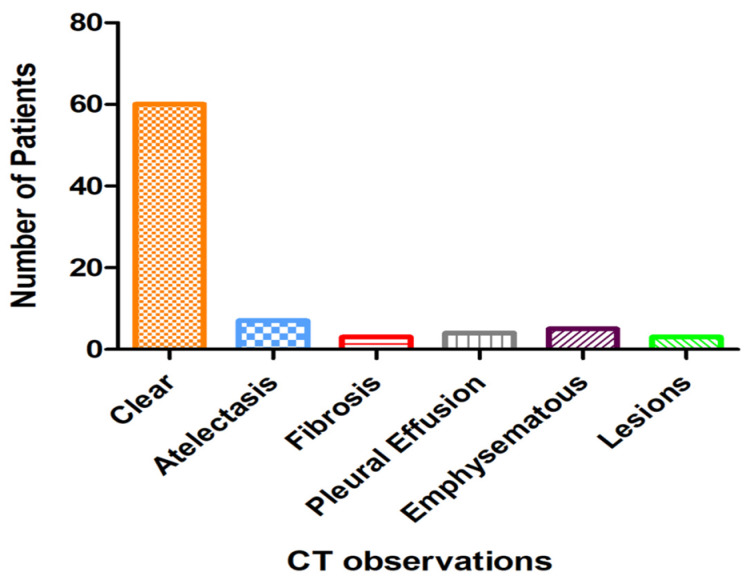
CT observations in patients with suspected pulmonary embolism (PE).

The most frequent observation was clear CT, with 60 patients showing no significant abnormalities. Atelectasis and emphysema were the next most common findings, although their prevalences remained relatively low. Other observations included fibrosis, pleural effusion, emphysematous changes, and lesions, each of which was noted in fewer patients, Figure 2.

These observations are instrumental in refining the interpretation of the V/Q SPECT results. They facilitate the identification of features that may appear indicative of PE in V/Q SPECT but do not correspond to PE when confirmed by CT scanning. This distinction is essential for enhancing diagnostic accuracy and avoiding unnecessary treatments.

The analysis revealed a sensitivity of approximately 63.2% and a specificity of approximately 93.2%, highlighting the enhanced specificity of V/Q SPECT CT. These findings suggest that this imaging technique is particularly reliable for accurately diagnosing pulmonary embolism, thereby reducing the likelihood of false-positive results.

## 4. Discussion

This study addressed the significant challenges in diagnosing pulmonary embolism (PE) given the difficulty in obtaining accurate and highly specific direct imaging of the affected area. PE can be fatal, particularly when initial symptoms are not promptly recognized or diagnosed. Survivors often suffer from subsequent organ damage due to oxygen deprivation, which affects vital organs such as the brain, heart, and lungs, potentially leading to pulmonary hypertension. Our results demonstrate that combining V/Q SPECT with ldCT enhances diagnostic accuracy and reduces radiation exposure compared to traditional methods. This finding aligns with recent guidelines from the European Society of Cardiology and the American College of Chest Physicians, which advocate improved diagnostic protocols. Furthermore, comparative studies corroborated our results, showing that V/Q SPECT combined with ldCT provides superior specificity and sensitivity. Addressing the questions that arise from these results, our study underscores the critical need for rapid and accurate diagnosis, which is supported by published literature that supports the adoption of advanced imaging techniques in clinical practice [10].

The European Association of Nuclear Medicine (EANM) guidelines endorse the use of V/Q SPECT for the diagnosis of PE. V/Q SPECT leverages the unique segmental anatomy of the pulmonary arteries to detect mismatches between ventilation and perfusion, which are indicative of PE [3]. According to Bajc (2019), PE is diagnosed if a V/Q mismatch in at least one segment or two subsegments aligns with the pulmonary vascular anatomy [3]. Despite its utility, V/Q SPECT has limitations, primarily owing to its lack of specificity. Other pathologies can produce segmental mismatches, resulting in false positives and potentially leading to unnecessary and harmful treatments.

Recently, hybrid imaging systems that integrate low-dose CT with V/Q SPECT have been developed to address these limitations. This study explored the efficacy of incorporating low-dose CT with V/Q SPECT for the diagnosis of PE. These findings suggest that the addition of low-dose CT enhances the specificity of V/Q SPECT. By providing detailed anatomical information, low-dose CT helps differentiate PE from other conditions that cause similar perfusion defects, thereby reducing the incidence of false positives and improving the overall diagnostic accuracy.

### 4.1. Ventilation/Perfusion Single-Photon Emission Computed Tomography with Low-Dose Computed Tomography: Specificity

Le Roux et al. indicated that using CT imaging alone as a substitute for Q SPECT could lead to a higher rate of overdiagnosis [7,10]. However, the results for combining CT with Q SPECT were less definitive because of the relatively small number of discrepancies between V/Q SPECT and V/Q SPECT CT. Our current study yielded similar findings, highlighting that certain cases diagnosed with Q SPECT might not be detected by CT alone and vice versa. Therefore, incorporating CT with Q SPECT reduces the risk of false positives and negatives.

Specificity is crucial when comparing diagnostic techniques, as high specificity helps to prevent overdiagnosis. In our study, V/Q SPECT had a specificity of 88%, whereas V/Q SPECT CT showed a higher specificity of 93%. This is consistent with the findings of Duan et al., who reported a sensitivity of 91% and a specificity of 94% using V/Q SPECT CT with a composite reference standard [6]. However, contrasting results were reported by Gutte et al., who found a specificity as low as 53% with V/Q SPECT CT, which was likely due to the lack of standardization in their study.

Certain thrombi, such as saddle thrombi, do not cause vascular occlusion and are therefore difficult to identify. These small thrombi are often clinically missed and, although unlikely to cause sudden death, require prompt diagnosis and treatment. Four patients in our study had small thrombi, which might have been missed if only V/Q SPECT had been used. V/Q SPECT CT can be particularly beneficial in identifying previously undiagnosed diseases.

Other studies have reported a specificity of 88% with V/Q SPECT alone and an increase to 93% with the addition of low-dose CT. Although our values are comparable, our study reported a much lower false-positive rate (5%) than 18% in other studies. This discrepancy may arise from different interpretations of segmental mismatches, highlighting the need for increased specificity of diagnostic techniques.

The interpretation of V/Q SPECT scans is subjective and can vary between users and institutions, underscoring the need for standardized interpretation protocols. Only one physician interpreted the results of our study, which may have introduced a bias. Discrepancies could arise if another physician interprets the results.

CTPA is a more favorable approach for detecting PE than V/Q SPECT, mainly because of the significantly shorter scan time (30 s for CTPA versus one hour for V/Q SPECT) [3]. Additionally, CT allows for direct visualization, whereas V/Q SPECT provides indirect visualization and prediction of PE. Although CTPA has high specificity (81–100%), our study noted that PE might be missed in cases where CT scans did not recognize it, which would not have occurred with V/Q SPECT scanning. This finding emphasizes the complementary roles of V/Q SPECT and CT in the diagnosing of PE [4,8,11].

### 4.2. Perfusion Single-Photon Emission Computed Tomography Substitution

The key objective of this study was to assess whether Q SPECT CT could serve as a viable substitute for V/Q SPECT CT in diagnosing PE. Using V/Q SPECT ldCT as the gold standard, it was found that if CT had been used as a substitute for ventilation, 21% of the patients would have been reported as positive for PE, with an 8% false-positive rate. This translates to a specificity of 60% for Q SPECT CT, which is significantly lower than that achieved by V/Q SPECT CT.

There is limited research on the replacement of V/Q SPECT CT with Q SPECT CT. However, some studies have reported a higher specificity for Q SPECT CT. For instance, one study reported a specificity of 80% for Q SPECT CT compared with 96% for V/Q SPECT CT. While these specificity values were higher than those obtained in the current study, it is important to note that the number of participants in the current study was considerably lower than that in other studies, which may account for the differences in results [12,13].

These findings highlight the need for more comprehensive studies with larger sample sizes to better evaluate the potential of Q SPECT CT as a substitute for V/Q SPECT CT. Despite some reports of higher specificity, current evidence suggests that V/Q SPECT CT remains superior in accurately diagnosing PE [7,13,14].

Integrating low-dose CT with V/Q SPECT for the diagnosis of pulmonary embolism (PE) offers multiple potential uses. Clinically, it enhances the diagnostic precision by reducing the number of false positives and unnecessary treatments. It provides a safer option for specific groups, such as pregnant women and those unable to use the traditional CTPA contrast medium. In emergency care, it expedites and refines the PE diagnosis, facilitating quicker and more accurate treatment decisions. This approach supports personalized medicine by incorporating patient-specific anatomical data for customized treatment strategies and fosters advancements in hybrid imaging, influencing future research in medical imaging and nuclear medicine [7,13,14]. These findings can inform healthcare recommendations and regulations and improve care quality and resource allocation.

Additionally, it enhances the training of radiologists and nuclear medicine specialists with advanced diagnostic tools and procedures. Finally, it supports health economic evaluations by demonstrating the cost-effectiveness of V/Q SPECT CT compared to other diagnostic methods, potentially influencing healthcare decision-making and reimbursement policies. Future research should focus on integrating advanced imaging technologies and artificial intelligence (AI) to enhance diagnostic accuracy and efficiency. Specifically, studies should explore the potential of AI algorithms in analyzing V/Q SPECT and ldCT images to improve diagnostic precision and reduce interpretation time. In addition, investigating the long-term outcomes of patients diagnosed with PE using these advanced techniques will provide valuable insights into their clinical utility and impact on patient care.

## 5. Study Limitations

The study limitations include the retrospective nature of data collection from a single hospital, which limits the generalizability of the findings. Although a systematic consultant reviewed the reports, the retrospective design may have resulted in the incomplete reporting of all aspects, including perfusion, ventilation, and CT findings. Additionally, the relatively small sample size of 92 patients further constrained the generalizability of the results. The exclusion of certain patient groups, such as pregnant women and those with comorbidities, while ensuring a focused assessment also limits the applicability of the findings to a broader population. Finally, potential observer bias due to the interpretation of scans by a single physician underscores the need for future studies involving multiple reviewers to enhance the robustness of the results.

## 6. Conclusions

The three main techniques suitable for diagnosing pulmonary embolism (PE) are V/Q SPECT, V/Q SPECT CT, and CTPA. Each method has its advantages and disadvantages.

V/Q SPECT and V/Q SPECT CT generally offer several advantages over CTPA, except for emergency out-of-hour settings. They provide lower radiation exposure and can be used in patients who cannot tolerate iodinated contrast agents. They also offer better sensitivity for the detection of smaller emboli. However, these techniques require more time and specialized equipment, making them less practical in acute emergency settings, where rapid diagnosis is crucial.

CTPA is favored in emergency settings because of its quick imaging time and ability to directly visualize the pulmonary arteries [15]. It provides high specificity and allows for the identification of alternative diagnoses, such as aortic dissection or pneumonia. However, CTPA involves higher radiation exposure and is not suitable for patients with renal insufficiency or allergies to contrast medium.

Certain patient groups, such as pregnant women and those with other health conditions, were excluded from the current study. To fully understand the benefits of V/Q SPECT, CT, including the evaluation of the full specificity of the technique, is essential. One way to achieve this inclusion is to use Q SPECT, which can be safely used in critically ill patients and pregnant women in their first trimester. Including these diverse patient groups would allow for a more comprehensive assessment of the efficacy and broader applicability of this technique.

Future research should focus on validating the efficacy of V/Q SPECT in diagnosing pulmonary embolism across diverse patient populations, including pregnant women and individuals with comorbidities, to ensure broader applicability and accuracy. Studies should explore the integration of advanced imaging technologies and AI to enhance diagnostic precision and reduce false positives. Additionally, longitudinal studies are necessary to assess the long-term outcomes and cost-effectiveness of V/Q SPECT in clinical practice. Collaborative multicenter trials can provide more comprehensive data, overcoming the limitations of single-center studies and facilitating the development of standardized diagnostic protocols.

## Figures and Tables

**Table 1:** Computed tomography protocol for V/Q SPECT CT.

Parameter	Value
kV	110
Effective mAs	16
Rotation time	0.6 s
Acquisition	16 × 0.6 mm
Slice collimation	0.6 mm
Slice width	5.0 mm
Feed/Rotation	9.6 mm
Pitch factor	1.00
Increment	5.0 mm
Kernel	B41s (standard), B90s (lung detail)
CTDIvol	1.28 mGy
Effective dose	Male: 0.68 mSv; Female: 0.86 mSv

## Data Availability

The data are available upon request from the corresponding author.

## References

[1] Maughan B.C., Jarman A.F., Redmond A., Geersing G.J., Kline J.A. (2024). Pulmonary Embolism. BMJ.

[2] Millington S.J., Aissaoui N., Bowcock E., Brodie D., Burns K.E.A., Douflé G., Haddad F., Lahm T., Piazza G., Sanchez O. (2023). High and Intermediate Risk Pulmonary Embolism in the ICU. Intensive Care Med..

[3] Bajc M., Schümichen C., Grüning T., Lindqvist A., Le Roux P.Y., Alatri A., Bauer R.W., Dilic M., Neilly B., Verberne H.J. (2019). EANM Guideline for Ventilation/Perfusion Single-Photon Emission Computed Tomography (SPECT) for Diagnosis of Pulmonary Embolism and Beyond. Eur. J. Nucl. Med. Mol. Imaging.

[4] Moore A.J.E., Wachsmann J., Chamarthy M.R., Panjikaran L., Tanabe Y., Rajiah P. (2018). Imaging of Acute Pulmonary Embolism: An Update. Cardiovasc. Diagn. Ther..

[5] Ameri P., Inciardi R.M., Di Pasquale M., Agostoni P., Bellasi A., Camporotondo R., Canale C., Carubelli V., Carugo S., Catagnano F. (2021). Pulmonary Embolism in Patients with COVID-19: Characteristics and Outcomes in the Cardio-COVID Italy Multicenter Study. Clin. Res. Cardiol..

[6] Duan J., Xie S., Sun H., An J., Li H., Li L., Grimm R., Voskrebenzev A., Vogel-Claussen J. (2023). Diagnostic Accuracy of Perfusion-Weighted Phase-Resolved Functional Lung Magnetic Resonance Imaging in Patients with Chronic Pulmonary Embolism. Front. Med..

[7] Le Roux P.Y., Robin P., Tromeur C., Davis A., Robert-Ebadi H., Carrier M., Le Gal G., Salaun P.Y. (2020). Ventilation/Perfusion SPECT for the Diagnosis of Pulmonary Embolism: A Systematic Review. J. Thromb. Haemost..

[8] Mortensen J., Gutte H. (2014). SPECT/CT and Pulmonary Embolism. Eur. J. Nucl. Med. Mol. Imaging.

[9] Treves S.T., Fahey F.H. (2022). Radiation Dose to Pediatric Patients From Radiopharmaceuticals. Semin. Nucl. Med..

[10] Le Roux P.Y., Robin P., Tromeur C., Davis A., Robert-Ebadi H., Carrier M., Couturaud F., Le Gal G., Salaun P.Y. (2018). SPECT V/Q for the Diagnosis of Pulmonary Embolism: Protocol for a Systematic Review and Meta-Analysis of Diagnostic Accuracy and Clinical Outcome. BMJ Open.

[11] Squizzato A., Rancan E., Dentali F., Bonzini M., Guasti L., Steidl L., Mathis G., Ageno W. (2013). Diagnostic Accuracy of Lung Ultrasound for Pulmonary Embolism: A Systematic Review and Meta-Analysis. J. Thromb. Haemost..

[12] Elojeimy S., Cruite I., Bowen S., Zeng J., Vesselle H. (2016). Overview of the Novel and Improved Pulmonary Ventilationperfusion Imaging Applications in the Era of SPECT/CT. Am. J. Roentgenol..

[13] Roach P.J., Schembri G.P., Bailey D.L. (2013). V/Q Scanning Using SPECT and SPECT/CT. J. Nucl. Med..

[14] Squizzato A., Venturini A., Pelitti V., Bellini B., Bernasconi M., Depalo T., Corso A., Riva N. (2022). Diagnostic Accuracy of V/Q and Q SPECT/CT in Patients with Suspected Acute Pulmonary Embolism: A Systematic Review and Meta-Analysis. Thromb. Haemost..

[15] Abuzaid M., Elshami W., Cavli B., Ozturk C., Almisned G. (2023). A Closer Look at the Utilized Radiation Doses during Computed Tomography Pulmonary Angiography (CTPA) for COVID-19 Patients. Radiat. Phys. Chem..

